# Cerebro-Spinal Meningitis: Its Infectivity and the Serum Treatment

**Published:** 1915-04-24

**Authors:** 


### CEREBRO-SPINAL MENINGITIS.

### Its Infectivity and the Serum Treatment.

The recent discussion on this subject at the Royal Society of Medicine was concerned chiefly with the epidemiology and bacteriology of the disease. At the same time there emerged certain points having interest for those engaged in general medical practice, and it may be convenient to present these in a brief summary. At the outset it may be stated with confidence that public apprehension and alarm have been unjustifiably excited by misleading rumours and reports. The number of cases has been greatly exaggerated, as also has the risk of infection. Occasional instances of the disease in children occur in London in the early part of every year, and they are well known in the Children's Hospitals, where, so little is the fear of extension, that the patients are usually treated in the general ward. The disease is never pandemic, as influenza, and even when it is prevalent it is uncommon to find more than a single example of it in one and the same household. Probably, for all practical purposes, there is little more risk of extension to doctors, nurses, and others who come into contact with the patient than exists in the case of pneumonia. The danger of infection seems to come principally from "carriers," in whom the germ is found in the naso-pharynx; these people may never develop meningitis themselves, but may convey the infection to others, in whom, presumably as resistance is less, typical evidences of meningeal inflammation may appear. Hence the advisability of taking swabs for bacteriological examination from the throats of those who have been in contact with patients. In this way "carriers" may be discovered and steps be taken to check their dangerous qualities. There is this further to be said on the favourable side—in comparison with other forms of meningitis spotted fever has a relatively hopeful prognosis. Few, if any, are the recoveries from tuberculous, pneumococcic, or septic meningitis, but cerebro-spinal meningitis spares from 25 to 50 per cent. of those whom it attacks.

In the diagnosis the practitioner must be prepared for wide departures from the classical description of the disease. At one extreme are "fulminant" cases, in which the patient dies so promptly that evidences of meningitis have not had time to develop themselves; at the other, are cases which continue for many weeks. Skin eruptions are by no means always present, and indeed the symptoms may be limited to headache, more or less retraction of the head, and some degree of Kernig's sign. Of great assistance is the examination of the cerebro-spinal fluid, which should never be omitted if there is even the least clinical suggestion of meningitis. Even to the naked eye the opalescent or milky appearance of the fluid is highly suggestive, and the microscope reveals polymorphonuclear cells and the meningococcus. Yet at times the fluid fails to display these characters, and it must be assumed that the diseased area is shut off by inflammatory exudates from the lower part of the cerebro-spinal space, which consequently yields a clear and normal fluid.

The results of treatment are somewhat confused by differences in the degree of virulence of the infection in different outbreaks. There is, however, reason to believe that the disease is not beyond the reach of therapeutic influence. Recently the intravenous or intramuscular injection of soamin has been advised, but those who remember the cases of optic atrophy which followed the use of this remedy in the treatment of syphilis some few years ago will hardly be encouraged to adopt it, and one of the speakers in the recent discussion strongly opposed it. With much greater confidence can the use of Flexner's serum be mentioned. In using this it has to be introduced into the cerebro-spinal canal. Lumbar puncture is performed in the usual fashion and fluid is withdrawn freely, as much as 30 cc. or more. Then a dose of 30 cc. of serum is introduced slowly by gravitation, and the process may be repeated at intervals of twenty-four hours. It is certain that in this way the activities of the micro-organisms may be neutralised, for after several doses the cerebro-spinal fluid may become clear, and when tested may be found to be sterile. An encouraging account from the clinical standpoint is given by Dr. Gardner Robb, of Belfast. In the three months which preceded the use of the serum there were forty-five cases, and of these thirty-seven were fatal, giving a mortality of 82 per cent. In the first thirty cases treated with the serum there were only eight deaths, that is a death-rate of 26 per cent., while, during the same period, of thirty-four patients not sent into hospital and not subjected to serum treatment twenty-nine died, a death-rate of 85 per cent. Another evidence of the value of the serum is the disappearance under its influence of the chronic cases. Formerly, in a proportion of the cases the patients remained in an unconscious state for weeks or months, were kept alive by nasal feeding, and yet in time died of emaciation. When the serum is used these experiences cease, and provided the patient does not die in the onset of the disease he rapidly improves and may be well in the course of three weeks or so. Early diagnosis and early use of the serum are important conditions if success is to be obtained. This to a very large extent means lumbar puncture, a method which ought to be much more generally practised. The recent experiences in London hardly give such a favourable impression of the serum treatment as those just quoted. There is reason to believe that in different outbreaks there are different "strains" of the meningococcus and that these need a corresponding variation in the serum. Attempts are being made on these lines, but the degree of success can hardly yet be stated. In the meantime it is certain that lumbar puncture and Flexner's serum have considerable value, and they ought to be utilised in every case, and this without delay.

